# Detection of Large Numbers of Novel Sequences in the Metatranscriptomes of Complex Marine Microbial Communities

**DOI:** 10.1371/journal.pone.0003042

**Published:** 2008-08-22

**Authors:** Jack A. Gilbert, Dawn Field, Ying Huang, Rob Edwards, Weizhong Li, Paul Gilna, Ian Joint

**Affiliations:** 1 Plymouth Marine Laboratory, Prospect Place, Plymouth, United Kingdom; 2 NERC Centre for Ecology and Hydrology, CEH Oxford, Oxford, United Kingdom; 3 California Institute for Telecommunications and Information Technology, University of California San Diego, La Jolla, California, United States of America; 4 Department of Computer Science, San Diego State University, San Diego, California, United States of America; 5 Mathematics and Computer Science Division, Argonne National Laboratory, Argonne, Illinois, United States of America; Centre for DNA Fingerprinting and Diagnostics, India

## Abstract

**Background:**

Sequencing the expressed genetic information of an ecosystem (metatranscriptome) can provide information about the response of organisms to varying environmental conditions. Until recently, metatranscriptomics has been limited to microarray technology and random cloning methodologies. The application of high-throughput sequencing technology is now enabling access to both known and previously unknown transcripts in natural communities.

**Methodology/Principal Findings:**

We present a study of a complex marine metatranscriptome obtained from random whole-community mRNA using the GS-FLX Pyrosequencing technology. Eight samples, four DNA and four mRNA, were processed from two time points in a controlled coastal ocean mesocosm study (Bergen, Norway) involving an induced phytoplankton bloom producing a total of 323,161,989 base pairs. Our study confirms the finding of the first published metatranscriptomic studies of marine and soil environments that metatranscriptomics targets highly expressed sequences which are frequently novel. Our alternative methodology increases the range of experimental options available for conducting such studies and is characterized by an exceptional enrichment of mRNA (99.92%) versus ribosomal RNA. Analysis of corresponding metagenomes confirms much higher levels of assembly in the metatranscriptomic samples and a far higher yield of large gene families with >100 members, ∼91% of which were novel.

**Conclusions/Significance:**

This study provides further evidence that metatranscriptomic studies of natural microbial communities are not only feasible, but when paired with metagenomic data sets, offer an unprecedented opportunity to explore both structure and function of microbial communities – if we can overcome the challenges of elucidating the functions of so many never-seen-before gene families.

## Introduction

DNA sequence based metagenomics has become a standard tool for the analysis of natural microbial communities in marine environments [1], [2], [3]. It involves the sequencing of random community DNA from environmental samples and subsequent determination of taxonomic and protein-encoding gene diversity. However, questions of how natural bacterial assemblages respond to perturbations in environmental conditions, are better answered by analysis of community mRNA than genomic DNA [4].

Historically, metatranscriptomic studies have involved either the use of microarrays [5] or mRNA-derived cDNA clone libraries [6]. These approaches have produced significant insight into the metatranscriptome of different communities but have limitations when exploring the diversity of natural communities. Firstly, a microarray only gives information about those sequences for which it was designed and it is usual to screen for gene sequences that are already known (e.g. from a gene-library or metagenomic sources). Secondly, although transcript cloning avoids this problem through the random amplification and sequestering of environmental mRNA fragments, it introduces other biases; e.g. any cloned transcripts that encode toxic products or titrates host DNA-binding factors will skew the relative abundance of sequences.

More recently, the first metatranscriptomic studies using high-throughput sequencing technology (pyrosequencing) have been published [7], [8], [9]. Two studies of soil communities have sequenced total RNA for the purpose of exploring both community structure, through the analysis of ribosomal RNA (rRNA), and community function, through the study of mRNA [7], [8]. The first study of a marine microbial community metatranscriptome focused on mRNA analysis and achieved an enrichment of ∼50% mRNA [9].

Ideally, if the study of mRNA is the prime purpose of a metatranscriptomic study, further enrichment is desirable. Here we present a study of a complex marine microbial metatranscriptome enriched to 99.92% mRNA [10]. Metatranscriptomes were generated from 4 samples taken at two time points in a replicated mesocosm (11,000 liters) study involving an induced phytoplankton bloom [10]. Pyrosequencing technology (GS-FLX pyrosequencer) was used to generate four metatranscriptomes and four corresponding metagenomes with an average sequence length of 215 bp from the middle and end time point in the phytoplankton blooms. This experiment provided an opportunity to obtain replicate samples to explore the use of this approach for detecting changes in the expression of genes over time. The primary focus of the mesocosm experiment was to study the response of marine microbes to the increase in ocean acidification that is resulting from dissolution of anthropogenic CO_2_ [10] and these results will be described in detail elsewhere. The immediate purpose of this study was to 1) demonstrate the feasibility of obtaining highly enriched samples of mRNA (>90%) from these communities, 2) determine whether differences in expression could be identified between time points of this controlled experiment [10], and finally 3) to determine what proportion of the most highly expressed genes using such a methodology might be novel.

## Methods

### Sampling, cDNA synthesis and sequencing

Water samples were obtained from a replicated mesocosm study (two treatments, each in triplicate) established in coastal waters of a fjord close to Bergen, Norway (60.27°N: 5.22°E). Each mesocosm contained 11,000 L of coastal water and two of the six mesocosms were sampled for this study. To induce the phytoplankton bloom, nitrate and phosphate were added. Water samples were taken at the peak and immediately following the collapse of the phytoplankton bloom from both a high CO_2_ and control mesocosm.

The nucleic acid extraction methodologies are briefly outlined in Gilbert *et al.* [10] but are fully described here. To isolate DNA and RNA, 15 L of water from each sample was filtered through a 140 mm diameter, 1.6 µm GF/A filter (Whatman), to reduce eukaryotic cell abundance and maximize the proportion of prokaryotic cells. This filtration took only 3 minutes and the filtrate was applied directly to a 0.22 µm Sterivex filter (Millipore) to allow rapid filtration of samples (<15 minutes per sample) to limited mRNA degradation. Following filtration, each Sterivex was pumped dry, frozen in liquid nitrogen and stored at −80°C until extraction. Total nucleic acid extraction was performed on each Sterivex using the method of Neufeld *et al.* [12]. Throughout the protocol nuclease-free plastic consumables and DEPC-treated water and reagents were used to limited degradation of mRNA. Following extraction, total nucleic acids were eluted in 200 µl of nuclease-free water.

For metagenomic analysis, 100 µl of the total nucleic acid extraction was purified for DNA by treatment with RiboShredder™ RNase (Epicenter) following manufacturer's instructions. Purified metagenomic DNA was quantified by nano-litre spectrophotometry, diluted with nuclease-free water to 500 ng µl^−1^ and then stored at −80°C until pyrosequencing.

For metatranscriptomic analysis, 100 µl of the total RNA was purified using the RNA MinElute™ clean-up kit (Qiagen); β-mercaptoethanol was added to the RLT buffer. Approximate RNA concentration was determined by nano-litre spectrophotometry and checked for rRNA integrity using an Agilent bioanalyser (RNA nano6000 chip). Average RNA concentration was 2.4 mg ml^−1^. The integrity of rRNA was demonstrated by highly defined, discrete rRNA peaks, with the 23S rRNA peak being 1.5–2 times higher than the 16S rRNA peak. Fully intact rRNA is essential for subtractive hybridization because degraded rRNA molecules will not be fully subtracted from the total RNA pool.

DNA contamination was removed from total RNA samples by treating with the Turbo DNA-free enzyme (Ambion). 75 µg of purified total RNA was applied to the subtractive hybridization method (Microbe Express Kit, Ambion) to remove rRNA from the mRNA. Purified mRNA was eluted in 25 µl of TE buffer (10 mM Tris-HCl pH 8.0, 1 mM EDTA) and was further purified with the MEGAclear™ kit (Ambion) to remove small RNAs and small contaminants. Purified mRNA was eluted in 10 µl of nuclease free water and stored at −80°C until further analysis. 0.5 µl of the purified mRNA was then checked using the Agilent bioanalyser for removal of genomic DNA and ribosomal RNAs. The mRNA concentration was estimated using the Agilent bioanalyser software to average 450 ng µl^−1^.

mRNA was estimated to be approximately 8% of total RNA isolated. 9.5 µl of the purified mRNA was then applied to a reverse transcription reaction using the SuperScript® III enzyme (Invitrogen) with random hexamer primers (Promega). The cDNA was treated with RiboShredder™ RNase Blend (Epicentre) to remove trace RNA contaminants. To improve the yield of cDNA, 1 µl of each sample was subjected to random amplification using the GenomiPHI™ V2 method (GE Healthcare) yielding approximately 4 µg of cDNA. GenomiPHI technology produces branched DNA molecules that are recalcitrant to the pyrosequencing methodology. Therefore amplified samples were treated with S1 nuclease using the method of Zhang *et al.* [13]. DNA and cDNA were nebulized to produce an average size of 500 bp, then cleaned with AMPure beads (Agencourt) and sequenced using the 454 Corporation's GS-FLX instrument at the NERC-funded Advanced Genomics Facility at the University of Liverpool (http://www.liv.ac.uk/agf/). Extraneous sequences resulting from >1 template molecule per picotitre well were removed from the datasets (**Table 1**) as they include exact duplicates and failed sequences that are replete with uncharacterized nucleotides. Metatranscriptomic and metagenomic data sets were deposited in NCBIs Gene Expression Omnibus (GEO, http://www.ncbi.nlm.nih.gov/geo/) and are accessible through GEO Series accession number GSE10119. All data is also deposited with the Short Reads Archive (NCBI) under accession number SRA000266. These datasets are also available with richer annotates in ISATAB format [14] compliant with the “Minimum Information about a (Meta) Genome Sequence” (MIGS) specification [15].

**Table 1:** Comparison of DNA and mRNA from samples from mid- and post-phytoplankton bloom.

	Mid-Bloom				Post-Bloom				Combined data		
	DNA-High CO_2_	mRNA-High CO_2_	DNA-Present Day	mRNA-Present Day	DNA-High CO_2_	mRNA-High CO_2_	DNA-Present Day	mRNA-Present Day	All DNA	All mRNA	All samples
Total size (Mbp)	47,289,282	**30,567,377**	30,991,689	**38,021,523**	59,316,369	**21,805,955**	68,187,679	**26,982,195**	205,784,939	**117,377,050**	323,161,989
Total No. of reads	209,073	**131,089**	134,915	**162,871**	344,216	**96,201**	304,020	**116,192**	992,224	**506,353**	1,498,577
Average length (bp)	226	**233**	229	**233**	172	**226**	224	**232**	207	**231**	215
% of rRNA genes^a^	0.33	**0.16**	0.31	**0.1**	0.17	**0.03**	0.24	**0.03**	0.25	**0.08**	0.16
Absolute number of unique nucleotide sequence clusters^b^	170,580	**65,717**	112,459	**67,283**	257,375	**9,349**	232,729	**10,703**	630,159	**133,447**	723,050
Normalized number of clusters^c^	86,791	**50,320**	84,096	**43,213**	86,996	**9,349**	87,112	**9,281**	n/a	**n/a**	n/a
Total number of reads in top cluster	12	**988**	23	**932**	19	**2,019**	20	**2,421**	36	**4860**	4866
Clustering: 1 sequence	141,340	**56,126**	94,386	**55,691**	200,569	**7,397**	183,028	**8,106**	437,149	**107,593**	494,604
2–9 sequences	29,232	**8,824**	18,072	**10,367**	56,729	**1,182**	49,681	**1,677**	191,822	**23,224**	224,198
10–99 sequences	8	**652**	1	**1,032**	77	**552**	20	**673**	1188	**2,011**	3,639
100+ sequences	0	**115**	0	**193**	0	**218**	0	**247**	0	**619**	609
SEED Subsystem hits	130,567	**75,884**	120,141	**83,076**	161,789	**16,545**	175,477	**18,315**	n/a	**n/a**	n/a
Total pORFs^d^	419,565	**284,665**	279,061	**345,502**	532,373	**237,187**	637,896	**289,951**	1,868,895	**1,157,305**	3,026,200
Unique pORFs at 95%^e^	358,705	**140,763**	242,317	**147,697**	435,876	**31,104**	515,266	**36,113**	1,340,241	**299,898**	1,571,348
Protein clusters^f^	321,839	**120,220**	223,888	**121,547**	382,762	**16,641**	452,754	**19,259**	1,083,644	**238,655**	1,228,601
Protein clusters^g^ with similarity to:	11	**296**	3	**468**	29	**516**	31	**589**	695	**1,131**	2,029
PFAM^h^	9	**19**	1	**23**	14	**56**	13	**63**	379	**76**	571
TIGRfam^i^	9	**0**	1	**0**	12	**0**	7	**0**	366	**1**	476
COG^j^	10	**2**	1	**0**	18	**0**	17	**0**	431	**6**	572
Number of novel Protein clusters^k^	0	**276**	2	**444**	9	**447**	12	**509**	202	**1026**	1287

Size, clustering and annotation data were generated by CAMERA. rRNA and subsystem hits were generated by SEED.

a Analysis of sequences against the Ribosomal Database Project II (RDP-II) and the European Ribosomal large subunit (LSU)dataset.

b Based on clustering at 95% identity over 80% length of a sequence and over 120 bp.

c For direct comparison of samples, individual rarefaction analysis, by R (http://www.r-project.org/) and the vegan package (http://cc.oulu.fi/jarioksa/softhelp/vegan.html), was used to estimate the number of clusters in each sample after adjusting sample size of each dataset to be equivalent to the number of reads in the smallest dataset (mRNA – High CO_2_).

d Partial Open Reading Frames (pORFs) from six reading frame translation from all reads using translation table 11, starting at the beginning of a read or first ATG after previous stop codon, ending at the end of a read, or at a stop codon and being at least 30 contiguous amino acids.

e Total pORF reads clustered at 95% identity of over 80% length of sequences.

f Clusters are identified using the representative sequences of each cluster from the 95% step to cluster at 60% identity of over 80% length of sequences.

g The dominant clusters (≥10 non-redundant sequences) with the exclusion of spurious pORFs.

h Protein families database.

i The Institute for Genomic Research protein database.

j NCBI clusters of orthologous groups database.

k With ≥10 non-redundant clustered sequences excluding spurious ORFs.

n/a – not analyzed.

### Clustering of DNA and mRNA and prediction of partial ORFs (pORFs)

Clustering analysis was performed on the raw reads and translated peptide sequences (see below) using the CD-HIT package [16]. The reads from all eight samples were clustered together with CD-HIT-EST program. Sequences were clustered if the identity was ≥95% (++ or +− strand) and the length of the alignment was ≥40 bp and ≥80% length of the shorter sequence. The clustering results show the internal structure of the combined dataset including number of non-redundant sequences, distribution of clustering, number of singletons, etc. The same analysis was applied to each individual sample by counting only the sequences from that sample. Results are shown in rows 5–11 in **Table 1**.

ORFs (including pORFs) were then predicted. Since genes cannot be reliably predicted from such short reads, we applied the methodology used in the Global Ocean Survey (GOS) study [2], [3], calling pORFs from all six reading frames. As the current study had overall shorter reads than the GOS study (average of 215 bp instead of 822 bp) pORFs had to contain at least 30 amino acids. In total 3,026,200 pORFs were detected. The approach of six reading frame translation can result in many non coding (shadow) pORFs, or spurious pORFs. However, this is less likely with short sequence data because the translations from non-coding frames are usually too short (due to random occurrence of stop codons) to rank as pORFs using our selected cut-off threshold.

The protein gene coding density, according to the most recent NCBI RefSeq database for microbial organisms (ftp://ftp.ncbi.nih.gov/refseq/release/release-statistics/RefSeq-release27.01062008.stats.txt), is about 0.25 million amino acid per 1 million base pairs (bp). This study, which obtained 162 million amino acids from 323 million bp of sequence, shows only 50% of these pORFs were spurious. Clustering of pORFs can further help to exclude spurious pORFs which are more likely to remain singletons.

The pORFs were clustered with two-step CD-HIT runs. At the first step, pORFs were clustered at 95% identity over 80% of sequence length in order to identify non-redundant sequences. The non-redundant sequences were further clustered at 60% identity, over 80% of sequence coverage, to find clusters of homologous pORFs or protein families (see row ^d, e, f^ in **Table 1**). Here, we only use the non-redundant sequences to count the size of each cluster so that the large clusters reported in row ^g^ in **Table 1** contain diverse sequences. The same clustering techniques were also applied to the data from the metatranscriptomic study of Frias-Lopez and colleagues [**9**] (**Table S1**).

### Dividing pORFs into ‘predicted, ‘spurious’ and ‘putative’

The clusters of pORFs were annotated by comparison to the PFAM database (http://PFAM.sanger.ac.uk/) by Hmmer, TIGRfam database (http://www.tigr.org/TIGRFAMs/) by Hmmer and the COG database (http://www.ncbi.nlm.nih.gov/COG/) by RPS-BLAST (reversed PSI-BLAST). All analyses were annotated with an expect value cut-off of 0.001. Hmmer analysis was performed in fragmental mode, and each hit also had to pass the TC score. The pORFs with significant matches to these reference databases were confirmed as genes, while the pORFs that overlapped with them from a different reading frame (the shadow pORFs) were deemed spurious pORFs. From this final analysis of the 3,026,200 pORFs, 494,253 could be confirmed as predicted proteins, 459,150 excluded as spurious pORFs, and the remainder (2,072,797) marked as “putative proteins”. The combined predicted and putative proteins were used for subsequent analysis.

### PCR detection of dominant orphan gene clusters in environmental DNA and mRNA

To validate the presence of highly expressed orphaned sequence clusters in the environment we randomly selected 27 of the most highly expressed nucleotide clusters. It was necessary to establish the presence of these sequences in both original DNA samples and cDNA samples to show they were not artefacts of cDNA amplification by GenomiPHI. To further cluster the 609 dominant nucleotide clusters (>100 sequences per cluster) for the purpose of designing PCR primers, all clusters were re-clustered at 95% identity over at least 40 base pairs. This allowed sequences with small 5′ or 3′ overlaps to be clustered together and increased the probability that the sequences assayed represented different transcripts. This reduced the number of dominant clusters from 609 to 85 (**Table S2**) and resulted in a significant increase in the number of clusters with more than 5,000 reads each. The maximum number of sequences in the largest cluster was 31,642 and 15 clusters now contained more than 5,000 reads. This provided a smaller pool of sequences for analysis and reduced the likelihood of amplifying similar sequences.

Primers were designed to screen 27 of these potential transcripts using the batch Primer3 online interface (http://probes.pw.usda.gov/cgi-bin/batchprimer3/batchprimer3.cgi), with the following conservative rules. First, we targeted the ‘representative’ sequence of each cluster (as opposed to the consensus sequence) to maximize the length of the query DNA sequence and avoid use of chimeric sequence that could have resulting from false assembly of the original 609 clusters. Second, we iteratively explored a range of parameters to find a rule that allows us to automatically create primers (no manual inspection required) for all 85 loci using a single set of optimality criteria that were as stringent as possible. In the end, we took the default parameters of the interface and optimized the following parameters: annealing temp (55°C), overall length of product (100 bp), primer size (20 bp), G+C content (50%) and minimum “maximum self-complementary”. Exact optimality criteria used for the selection of each batch of primers is available from the authors.

Individual transcript sequences were amplified by PCR from the environmental DNA used for the metagenomic analyses (co-extracted with the mRNA used for the metatranscriptomic approach), purified mRNA prior to RT-PCR and cDNA prior to GenomiPHI amplification. Each of the 54 primers (http://nebc.nox.ac.uk/nebcfs/public/Joint/metatranscript_primers.xls) were diluted to a working concentration of 10 pmol µl^−1^. Approximately 10 ng of environmental DNA, cDNA or mRNA was added to a 25 µl PCR reaction with final concentrations of 1×PCR buffer (Promega), 2.5 mM MgCl_2_, 0.2 mM deoxynucleoside triphosphates (Invitrogen), 0.4 pmol of each primer, and 1 unit of *Taq* DNA polymerase (Promega). Negative controls used were *Escherichia coli* K12 genomic DNA and sterile water. Reactions were cycled with a PTC 1000 thermal cycler (MJ Research) using the following conditions; 94°C for 2 minutes, 30 cycles of 94°C for 1 minute, 55°C for 1 minute, 72°C for 2 minutes, and a final extension of 72°C for 10 minutes. Products were visualised by agarose gel electrophoresis (1.8%).

## Results and Discussion

We demonstrate the feasibility of conducting metatranscriptomic studies on RNA samples highly enriched for mRNA from natural microbial communities. This is the first time such a high level of enrichment has been achieved in a metatranscriptomic study (**Table 2**). Eight samples, four DNA and four mRNA, were processed producing a total of 323,161,989 bp (117.4 Mbp of mRNA and 205.7 Mbp of DNA). This exceeds previously published metatranscriptomic studies because of the inclusion of replicated samples. By further contrast, this is equivalent to 5.1% of the total bp sequenced, and 19% of the number of reads of the recent Global Ocean Survey (GOS) sequencing effort [2]. Here we present an analysis of these data that confirms the high level of enrichment for mRNA and the high levels of assembly of mRNA sequences compared to the DNA of the metagenomes; we also speculate on the potential coverage of the natural metatranscriptome sampled and discuss potential biases introduced by this methodology and provide evidence against the large-scale generation of mosaics and artefacts by the use of GenomiPHI amplification. We then discuss the proportion of these mRNAs that match protein databases, discuss the most abundant ‘known’ clusters, and compare the metatranscriptome with the metagenome and with the first published study of a marine metatranscriptome [9].

**Table 2:** Comparison of methods described by current manuscript with the three most recent methods for analysing microbial metatranscriptomes.

	Leininger et al [7]; Urich et al [8]	Frias-Lopez et al. [9]	Gilbert et al [10] (and this study)
**Habitat**	Soil (Nutrient-poor, sandy-soil)	Marine (oligotrophic ocean)	Marine (eutrophic coastal waters)
**Total biological samples**	1 (1 metatranscriptome)	1 (1 metatranscriptome, 1 metagenome)	4 (4 metatranscriptomes, 4 metagenomes)
**Total DNA/RNA (Millions bp)**	∼25.32	∼60.1	∼323.2
**RNA purification methodology**	Griffiths et al [11] method from 6 g of soil.	mirVana RNA isolation kit (Ambion) from 1 L of sea water	Neufeld et al, [12] method and MinElute RNA cleanup (Qiagen) from 15 L of seawater
**mRNA isolation and amplification methodology**	N/A *	mRNA amplification using MEssageAmp II-Bacterial kit (Ambion)	MicrobeExpress and Megaclear kit (Ambion). GenomiPHI amplification (GE Healthcare)
**RNA sequencing**	GS20-pyrosequencing	GS-20 pyrosequencing	GS-flx pyrosequencing
**Average length**	98 bp	112 bp	215 bp
**Yield of mRNA sequences**	8.2%	47.1%	99.9%
**Yield of orphaned sequences**	22% (60% of mRNA assigned tags)^1^	89.5%^2^	87%^2^

1 based on hits to nucleotide sequences using the MG-RAST Seed database.

2 based on hits to potential open reading frames using the PFAM, TIGRfam and COG protein databases.

* Not performed, rRNA and mRNA expressly sequenced together to examine both community structure and function.

### Determining the proportion of ribosomal RNA remaining in mRNA metatranscriptomic samples

Both DNA and mRNA sequences were analyzed using the publicly-available SEED MG-RAST (Metagenome Rapid Annotation using Subsystem Technology, http://metagenomics.theseed.org [17], [18]), which compares inputted sequences against a database of metabolic systems from selected organisms. Taxonomic information for the metagenomes within SEED was obtained by comparison against three 16S rDNA databases (the Ribosomal Database Project II (RDP), Greengenes, and the European Ribosomal Database). Although rRNA comprises approximately 80–90% of total RNA in a typical bacterium [19], it averaged only 0.08% of the total number of sequences in the four combined cDNA libraries (**Table 1**). The purification was far more efficient than would be predicted for the methodology and capture-probe range of the Microbe Express kit (Ambion) used for the subtractive hybridisation of rRNA. This could be because the 16S rRNA probes used in the subtractive hybridisation technique may hybridise to a more significant proportion of the community than previously considered. While this might lead to a more substantial removal, it cannot explain the near-complete removal seen in this study. A second more likely possibility is that the multiple displacement amplification approach (GenomiPHI) used to amplify the available mRNA, inefficiently amplified rRNA due to its inherent secondary structure that could have inhibited the reaction (GE Healthcare technical services communication). Both of these options should be further tested.

### Comparisons of homology between datasets

To determine the similarity of each dataset to each other, total nucleic acids between each database and total partial ORFs (pORFs) between each database were compared to provide an indication of the number of homologous sequences shared between each pair of datasets (**Table S3**). This demonstrated that each DNA dataset shared approximately 10% to 25% of the nucleic acid sequences and 20% to 33% of the pORF sequences. This suggests that the majority of sequences within each group were unique (singletons) to each dataset; a similar result was seen in the Global Ocean Survey when the metagenomes of different regions were compared [2]. The comparison between mRNA datasets showed a clear delineation between mid-bloom and post-bloom, with mid-bloom mRNA sharing ∼50% of their nucleic acid transcripts and post-bloom sharing >95% of their nucleic acid transcripts. This result was consistent when the datasets were compared between time points, with ∼50% of mid-bloom transcripts being homologous with ∼90% of post-bloom transcripts (**Table S3**). We postulate below that this difference could be due to an over-abundance of viral transcripts in the post-bloom environment causing the metatranscriptomes to become more homogenous.

It was expected that the metatranscriptome from the post-bloom environment would be more similar to the metagenome from the post-bloom environment than the mid-bloom samples. The comparison clearly demonstrates this (**Table S3**). The mid-bloom metagenomes also had greater homology to the mid-bloom metatranscriptomes than the post-bloom metatranscriptomes.

### Clustering of DNA and mRNA sequences confirms higher levels of assembly of mRNAs and differences between time points

To determine the possible level of assembly of sequence clusters, total DNA and mRNA sequences were analyzed using a metagenomic sequence analysis pipeline developed at CAMERA (Community Cyberinfrastructure for Advanced Marine Microbial Ecology Research and Analysis [20]) (access to this pipeline can be arranged by contacting the corresponding author). The number of unique sequences was calculated by clustering un-assembled sequence reads as described in the Materials and Methods [16].

As shown in **Table 1** an average of 79% of the DNA-derived metagenome sequences from both mid- and post-bloom samples were unique (singletons). This confirms the low level of coverage of the genomes in this sample and the high diversity. In contrast, the mRNA-derived sequences showed much higher levels of clustering, with an average of 45% of the sequences from the mid-bloom (time point 1) and only 9.5% of the post-bloom sequences (time point 2) being unique (**Table 1**) (calculated by dividing the total number of unique sequence clusters by the total number of sequence reads). Strikingly, only five of the 609 largest nucleotide-level mRNA clusters (those with ≥100 sequences) had an observed match in any of the DNA metagenomes. This low level of homology between mRNA sequences and the community DNA metagenome was previously noted [9] and is expected given the sparse sequence coverage for the much larger metagenome. Alternatively, this could be an overestimate of the lack of homology as it is possible for any of the RNA clusters to actually come from the same transcript. For example, an mRNA cluster from the first 25% of a given gene (average gene length ∼950 bp [21]) would appear to have no match even if the DNA library captured the other 75% of the gene. Unfortunately, there is no way to resolve this issue given the small size of sequences currently generated with pyrosequencing methodology.

To directly compare the level of assembly (diversity) in the eight samples, individual rarefaction was used to normalize the number of clusters to an equivalent sampling effort (i.e. that of the smallest sample) (**Table 1**). The number of clusters in all four DNA samples was surprisingly uniform and was double the number of clusters from the mid-bloom and nine times from the post-bloom mRNA samples. These results show that the metatranscriptome is smaller than the metagenome, assembles better, and that the expression of genes is different for mid-bloom and post-bloom communities. Of these an average of 0.23% of mid-bloom and 2.3% of post-bloom transcript clusters included more than 100 sequences. In other words, the transcription profile became more homogenous in the post-bloom situation.

Based on this clustering, we compared the total number of clusters found to the total number expected within a given water sample. To generate a rough estimate of potential metatranscriptome coverage, we used the approach of Poretsky *et al.* [6] to estimate that each water sample contained ca. 80,000 unique transcripts. This estimate is based on the observed number of dominant taxa and bacterial abundance (data not shown). This is the same order of magnitude as the number of unique mRNA sequences identified (**Table 1**) suggesting that this study may have achieved a reasonable coverage of the community metatranscriptome (in comparison, the metagenomes were vastly under-sampled). This is clearly an upper estimate, and given that the top 609 nucleotide clusters could be collapsed with less conservative clustering criteria into 85 larger clusters (see Materials and Methods), the actual number of transcripts could be 7-fold or more lower. Indeed the real value could be even lower, since one functional transcript may be coded for by more than one cluster (**Table 3**). We have previously shown this for another gene, *phnA*, that encodes phosphonoacetate hydrolase; the *phnA* from one organism had twelve hits within the metagenomic data, which were spread out over the gene [10]. Using the clustering methodology outlined here, this method would have identified this one gene as belonging to six different clusters due to overlap between the 12 sequences.

**Table 3:** Top ten most abundant identifiable transcripts identified from pORF clustering.

Rank	COG ID	No. of Seqs (nr)	No. of clusters	Annotation	TIGRfam ID	No. of Seqs. (nr)	No. of clusters	Annotation	PFAM ID	No. of Seqs. (nr)	No. of clusters	Annotation
1	COG0209	464 (149)	12	Ribonucleotide reductase, alpha subunit	TIGR02505	526 (190)	13	ribonucleoside-triphosphate reductase, adenosylcobalamin-dependent	PF02407	27147 (815)	44	Putative viral replication protein
2	COG0443	330 (156)	11	Molecular chaperone	TIGR02348	359 (158)	11	chaperonin GroL	PF00910	15101 (595)	28	RNA helicase
3	COG0459	359 (158)	11	Chaperonin GroEL (HSP60 family)	TIGR02350	330 (156)	11	chaperone protein DnaK	PF00005	372 (212)	18	ABC transporter
4	COG0376	96 (46)	9	Catalase (peroxidase I)	TIGR01369	214 (108)	9	carbamoyl-phosphate synthase, large subunit	PF00004	326 (149)	13	ATPase family associated with various cellular activities (AAA)
5	COG0458	214 (108)	9	Carbamoylphosphate synthase large subunit	TIGR02188	236 (120)	9	acetate–CoA ligase	PF00012	330 (156)	11	Hsp70 protein
6	COG5265	236 (126)	9	ABC-type transport system involved in Fe-S cluster assembly, permease and ATPase components	TIGR00630	224 (103)	8	excinuclease ABC, A subunit	PF00118	359 (158)	11	TCP-1/cpn60 chaperonin family
7	COG0086	210 (96)	8	DNA-directed RNA polymerase, beta' subunit/160 kD subunit	TIGR00936	257 (118)	8	adenosylhomocysteinase	PF00006	292 (122)	10	ATP synthase alpha/beta family, nucleotide-binding domain
8	COG0178	224 (103)	8	Excinuclease ATPase subunit	TIGR02013	219 (82)	7	DNA-directed RNA polymerase, beta subunit	PF00009	399 (148)	10	Elongation factor Tu GTP binding domain
9	COG0499	257 (118)	8	S-adenosylhomocysteine hydrolase	TIGR02506	267 (78)	7	ribonucleoside-diphosphate reductase, alpha subunit	PF02867	326 (101)	9	Ribonucleotide reductase, barrel domain
10	COG0085	219 (82)	7	DNA-directed RNA polymerase, beta subunit/140 kD subunit	TIGR01242	178 (84)	6	26S proteasome subunit P45 family	PF00501	228 (116)	8	AMP-binding enzyme

This table only includes the pORF clusters with >10 non-redundant sequences. No. of Seqs. refers to the number of sequences which contribute to that cluster, in brackets are the number of non-redundant sequences which contribute to that cluster.

### Evidence against potential biases in detecting naturally occurring mRNA clusters introduced by GenomiPHI

There are two key biases that might be introduced using the methodology presented here. Firstly, the time required to concentrate the community by filtration is longer than the half-life of mRNA, but this is true of most methods used to analyze the metatranscriptome of aquatic samples [6]. Recent studies however, have used smaller volumes (e.g. ∼1 L [9]), and the current methodology would still be effective using these smaller volumes. However, in the current study this methodology was run concomitantly with other analyses that required a significant amount of DNA, e.g. fosmid library production [10].

Secondly, amplification of cDNA using GenomiPHI could introduce artefactual sequences (although evidence of such a bias for transcriptome amplification does not exist [22]). Such artefacts could include mosaic or artefactual sequences that could explain the large number of orphan transcripts found in this study. We therefore performed four types of subsequent analyses to attempt to validate these clusters.

Firstly, to generate empirical evidence of the presence of these clusters in the original water samples and to test for chimeras, a PCR analysis was performed that targeted 27 of the most highly expressed orphan clusters. PCR reactions were performed on 1) the original environmental DNA preparations, 2) unamplified cDNA and 3) mRNA (this was a negative control, since it is DNA-free). Amplification products were detected for all 27 selected target sequences in at least one of the environmental DNA samples (**Table S2**). None of the sequences could be detected in any of the 4 mRNA samples (negative controls) confirming an absence of contamination of DNA. All 27 transcripts were found in all four cDNA samples. For the mid-bloom time points, 12 and 11 of the transcripts respectively were identified in the high CO_2_ and control environmental DNA samples **(** **Table S2**). Some, but not all, of these transcripts were of lower abundance when normalized to sequencing effort (data not shown).

Secondly, this is the first published metatranscriptomic study to include biological replicates (**Table 2**) making it possible to compare observed transcripts generated from independent samples using the same methodology. Of the four metatranscriptomes analyzed, transcript clusters showed similar abundance in *both* peak bloom samples and *both* post bloom samples (**Table 1** **,** **Table S2**). Since all four metatranscriptomes were generated using the same mRNA enrichment methodology, this level of observed similarity of abundant transcripts would not be expected by chance and provides strong evidence that the difference seen *between* time points in both the treatment and control samples are due to biological differences in the composition of the community within the bloom (**Table S2**).

**Figure 1:**
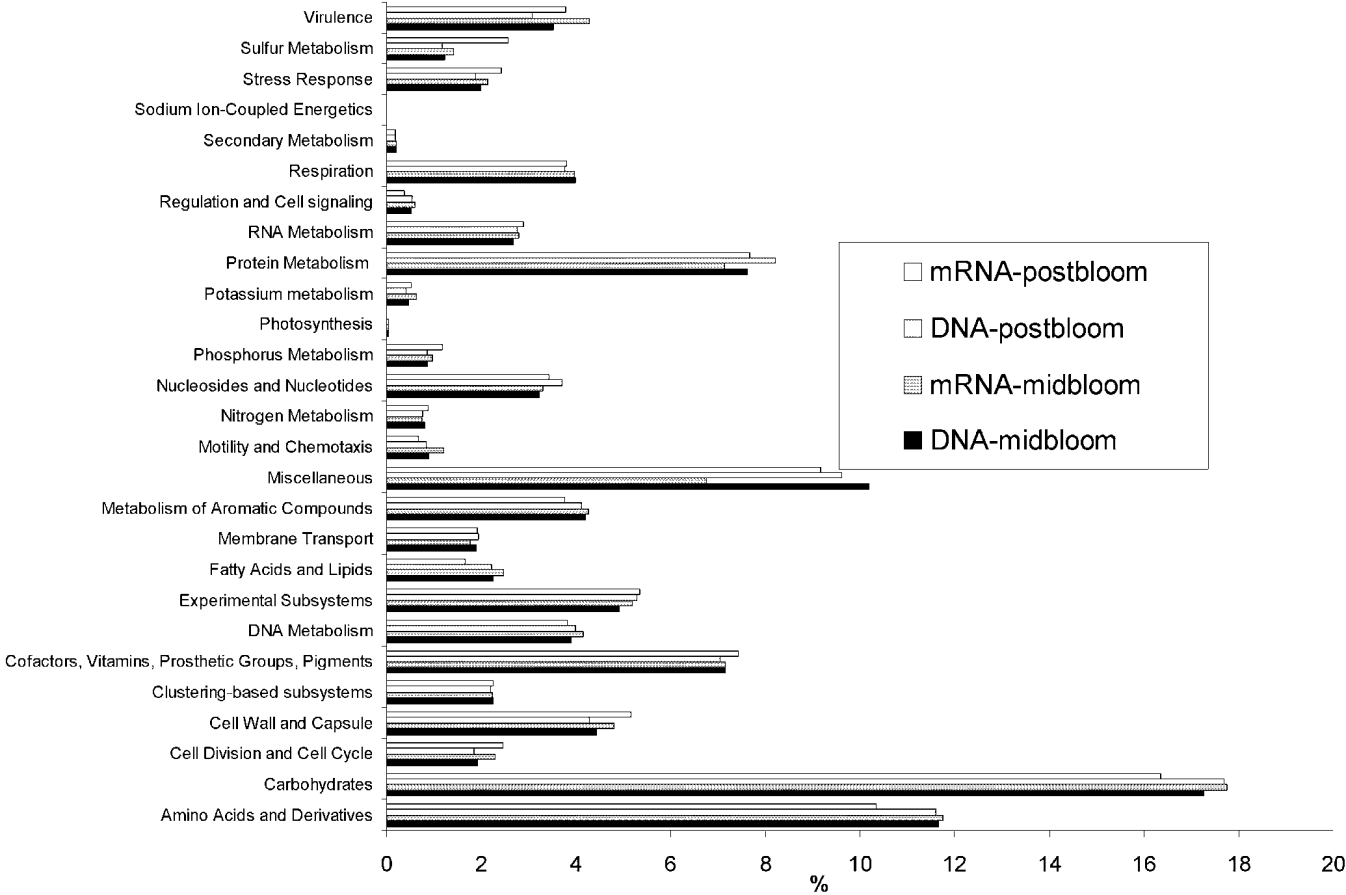
Relative abundance of sequence types identified for each sample. Number of sequences per metabolism subsystem were normalised to sequencing effort for each sample and then relative abundance for each was calculated as a percentage.

Thirdly, we compared the functional profiles of the metagenomes and metatranscriptomes (**Fig. 1**). All eight data sets were annotated using similarity matching against SEED subsystems [17]. While this approach only validates transcripts with observable homology to genes in known subsystems, it still shows that the metatranscriptome functional profile does not significantly differ from that of the metagenome (one-way Anosim R = 0.271, *p* = >0.05). For this analysis, the number of sequences with significant identity to each metabolic gene in a functional category in the SEED subsystem database were normalised to the sequencing effort for each sample (**Fig. 1**) and sequences which could not be annotated in this way were not included.

Fourthly, we compared the level of assembly and novelty between our four mRNA and DNA samples and that of the only previously published metatranscriptomic study of a marine microbial community [9]. All samples were translated in all six reading frames into contiguous peptides of at least 30 amino acids without a stop codon, spurious pORFs were removed, leaving 2,567,050 predicted or putative pORFs (See Materials and Methods, **Table 1**). These pORFs were clustered to assess the diversity of function from each sample, and were compared against known databases to provide basic annotation of known proteins and potential identification of novel pORFs. As shown in **Table 1**, the majority of the highly clustered (≥10 non-redundant sequences per cluster) transcripts (∼94% mid-bloom and ∼87% post-bloom) were novel clusters that may represent uncharacterized proteins.

The mRNA samples from the current study yielded 1∼2 orders of magnitude more novel protein clusters than their corresponding DNA samples when normalized to size (**Table 1**). Surprisingly, this high level of diversity was actually exceeded by the previously published Frias-Lopez study [9]. Clustering of that data according to the same criteria showed that ∼98% of metatranscriptomic sequences were unique (**Table S1**). To directly compare the annotation of pORFs for this study with that of the Frias-Lopez study [9], we applied the same clustering techniques for the translated proteins to their raw data (**Table S1**). A total of 1,826 pORF clusters containing >10 non-redundant sequences were found (DNA, rRNA and mRNA) and 865 pORF clusters remained after all rRNA clusters were removed. If the values for novel protein clusters are normalised to sequencing effort, we see that the Frias-Lopez study identified 1.8× the number of novel protein sequences per sequencing effort when compared to the current study. This phenomenon can be partially explained by the differences in read lengths between the studies (**Table 2**), as longer read lengths are more likely to be positively annotated than shorter read lengths [23], [24].

### Differences between sequence abundances in metatranscriptomes and metagenomes

**Figure 2:**
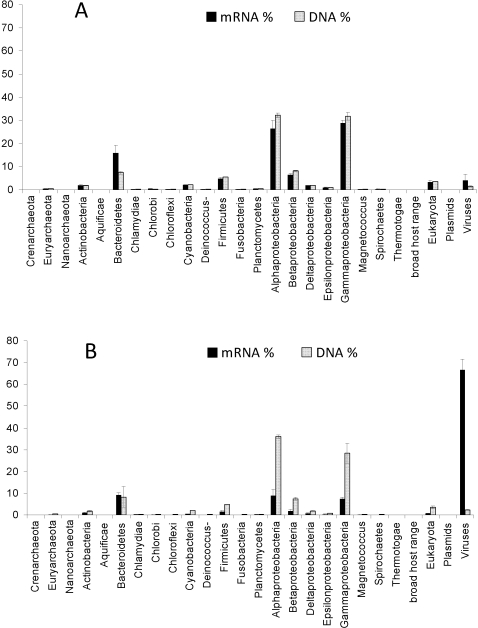
Percentage taxonomic affiliation of sequences identified in each dataset by BLAST against the SEED database. A – community at peak of the phytoplankton bloom (±1 SD). B - community after the phytoplankton bloom (±1 SD). Standard deviations are calculated from comparison of the different treatments. Data shown are for the high CO_2_ treatment.

The benefits of applying both metatranscriptomic and metagenomic analysis to the same biological samples include the potential to detect differential expression of mRNAs (function) between communities under different environmental conditions, while the metagenome (DNA) can also provide a frame-of-reference for the total potential of the community metatranscriptome. Using the proportion of DNA and mRNA sequences that had homology to known proteins, we were able to make phylum-level taxonomic assignments using annotations from the SEED databases [18] (**Fig. 2**). Comparison of the 4 DNA and 4 mRNA samples shows them to be significantly different in taxonomic composition (by one-way Anosim analysis, R = 0.385, *p*<0.03). Despite this, comparisons of all subsets of the data failed to reveal any significant differences (perhaps due to small sample sizes – data not shown). This suggests that changes seem in mid- and post-bloom time points are due more to changes in particular genes within taxa, than large-scale changes in the abundances of phyla-level taxonomic groups. Some potential qualitative changes can be seen within these patterns that may contribute to this significant difference between DNA and mRNAs including an increased number of transcripts from the *Bacteroidetes* phylum (an important group in macromolecule degradation) during the mid-bloom sample compared to its proportion of the same sample of DNA (Bacteroidetes was only the 4^th^ most abundant metagenomic group but had the 3^rd^ highest transcriptional activity) (**Fig. 2**).

### The most abundant ‘known’ transcripts found in the metatranscriptome and metagenome

The most abundant ‘known’ pORF clusters included a large number of housekeeping genes. All pORF clusters with >10 non redundant sequences were annotated by comparison to the PFAM, TIGRfam and COG databases (**Table 3**). Whilst the PFAM annotations yielded significant numbers of viral proteins, viral sequences were absent from both the TIGRfam and COG annotations (viral annotations discussed in next section).

Among the most abundant sequences with annotations are stress-induced chaperonin proteins (**Table 3**), which are potentially expressed in response to the low pH or high CO_2_ concentration stress found in the ocean acidification mesocosm samples. This is corroborated by the distribution of sequences with ∼60% (1.4×increase) of the chaperonin transcript sequences coming from the high CO_2_ mesocosms. This is mirrored by the metagenomic data in which ∼55% (1.2×increase) of the chaperonin gene sequences are found in the high CO_2_ environment. However, it is possible that these proteins are induced when the bacteria are being filtered, and hence this could be an artefact caused by the sampling procedure; using smaller starting volumes should alleviate this. Neither of these proteins were identified as being abundant in the dominant pORF clusters (>10 non-redundant sequences) from study by Frias-Lopez *et al.* [9] which utilised only 1 L sampling volumes and may have reduced stress on the bacteria by reducing the filtration time.

A range of proteins considered to be ubiquitous in cellular processes also ranked among the most abundant sequences that could be annotated. These included ribonucleotide reductase proteins (COG0209, TIGR02505, TIGR02506, PF02867), which were matched in all 3 reference databases (**Table 3**), as were proteins involved in ABC transporters, ATPase activity and AMP-binding (COG5265, TIGR00630, PF00004, PF00005, PF00006, PF00501). RNA polymerases (COG0085, COG0086, TIGR02013) were only assigned by the COG and TIGRfam annotations. Other abundant pORF clusters (>10 non-redundant sequences) encoded catalases/peroxidises (COG0376), carbamoylphosphate syntheases (COG0458, TIGR01369), excinucleases (COG0178), S-adenosylhomocysteine hydrolase (COG0499, TIGR00936), acetate-CoA ligases (TIGR02188), 26S proteasome subunit P45 family protein (TIGR01242), and elongation factor Tu GTP binding domain (PF00009) (**Table 3**).

### Abundant viral sequences in the post-bloom time point samples may contribute to the large number of orphan transcript clusters found

At the end of any phytoplankton bloom, a substantial increase is expected in the number of expressed viral transcripts. This was observed in our post-bloom mRNA samples, in which transcripts with viral homologues were on average 24.5 times more abundant than viral DNA sequences (**Fig. 2**). While free viruses particles would pass through the 0.22 µm filters used, and would therefore have low sequence abundance in the post-bloom samples, infected cells would be expected to have overwhelming viral gene expression during lytic growth (**Fig. 2**). The large increase in viral transcription occurred immediately after a substantial increase in bacterial abundance following the phytoplankton bloom (data not shown).

The high expected abundance of viruses in the post-bloom environment suggests that many of the unknown predicted proteins maybe of viral origin [3]. This is supported by the annotation of the dominant pORF clusters (with >10 non-redundant sequences, **Table 3**) and **Fig. 2**. The most abundant sequence that could be annotated was PF02407, Putative Viral Replication Protein, and the second most abundant was PF00910, RNA helicase, which is thought to be involved in viral infection (**Table 3**). These sequences comprise 7.7% and 5% respectively of the dominant clusters that can be annotated by comparison to the PFAM database (12.7% in total).

Furthermore, these two proteins are more abundant in the post-bloom environment, with ∼86% of these transcripts being found only in the post-bloom samples. Interestingly, only a single homologue for PF02407 was found in the post-bloom metagenomes. This not only confirms the results seen in **Fig. 2**, but also underscores a clear case of the biological significance of observed differences in the ratios of transcripts and their DNA sequences.

### Further validation of metatranscriptomes and metagenomes by direct comparison to an oligotrophic ocean metatranscriptome

Both the validity and nature of mRNA transcripts from this experiment were explored by direct comparison with the Frias-Lopez data set (**Table 2**) [9]. There was some overlap of sequences, including both house-keeping genes and a few of the most highly expressed novel orphan clusters. But the analysis also highlights the extensive diversity between these samples which, while both taken from the marine environment, came from two distinct marine habitats (**Table 2**).

Specifically, comparisons were generated using BLASTN (**Table 4**) for 3 versions of the two data sets: 1) total sequences, 2) representative ntDNA sequences from each nucleotide cluster and 3) representative sequences from each pORF cluster. Both mRNA and DNA sequences were compared separately. Values for the Frias-Lopez cDNA following removal of the rRNA sequences were also used for comparison. The most abundant clusters of the current study were also compared to the Frias-Lopez full mRNA and DNA datasets (**Table 5**).

**Table 4:** BLASTN comparison of total nucleic acids, representative sequences from nucleic acid clusters and representative sequences from pORF clusters from this study and the Frias-Lopez study [9].

		Gilbert et al [10] and current study			
		*DNA, DNA nuc-clusters, DNA pORF clusters*		*mRNA, mRNA nuc-clusters, mRNA pORF clusters*	
**Frias-Lopez et al ** [**9**]	*DNA*	44261(10.7)	102637 (10.3)	19359 (4.67)	56835 (11.2)
	*DNA nuc-clusters*	35575 (10.6, **8.6**)	59918 (9.5, **6**)	15564 (4.65, **3.75**)	11698 (8.8, **2.3**)
	*DNA-pORF clusters*	59774 (15, **14.4**)	40002 (3.7, **4**)	21302 (5.5, **5.1**)	17672 (7.5, **3.5**)
	*mRNA*	64609 (52.4)	18602 (1.9)	58123 (45)	2680 (0.53)
	*mRNA (rRNA removed)*	15179 (24.1, **11.8**)	15598 (1.57, **1.6**)	13121 (20.8, **10.2**)	2162 (0.43, **0.42**)
	*mRNA nuc-clusters*	27094 (38.7, **21.1**)	10689 (1.7, **1.07**)	22289 (31.9, **17.4**)	1942 (1.45, **0.38**)
	*mRNA nuc-clusters (rRNA removed)*	8338 (19, **6.5**)	9631 (1.5, **1**)	6171 (14, **5**)	1624 (1.2, **0.3**)
	*mRNA-pORF clusters*	4330 (9, **3.4**)	5484 (0.5, **0.55**)	2372 (5, **1.8**)	1736 (0.7, **0.34**)

For each comparison two values are given, the first value is the percentage of Frias-Lopez data which is homologous to data from the current study; the second is the percentage of data from the current study which is homologous to the Frias-Lopez data. Comparisons were performed using BLASTN with the current studies dataset as reference database, and the Frias-Lopez dataset as the query. The (-b –v) parameter in BLASTN was set to 40,000. For every query sequence, every similar sequence in the reference dataset is identified. Sequences from both datasets that meet the criteria of an E-value <0.001 were included. Percentage values in parentheses are calculated by dividing each value by the total number of sequences/representative sequences for each dataset. For the Frias-Lopez data: Total DNA – 414,323, Total DNA nuc-clusters – 334,940, Total DNA pORF clusters – 390,599, Total mRNA – 128,234, Total mRNA (rRNA removed) - 63,111, Total mRNA nuc-clusters – 69,948, Total mRNA nuc-clusters (rRNA removed) - 43,948, Total mRNA-pORF clusters – 46,703. For the Gilbert data: Total DNA – 992,224, Total DNA nuc-clusters – 630,159, Total DNA pORF clusters – 1,083,644, Total mRNA – 506,353, Total mRNA nuc-clusters – 133,447, Total mRNA pORF clusters – 238,655. Percentage values in bold are normalised by divided each value through the Total DNA or Total RNA for the relevant study. Nuc-cluster refers to nucleotide clusters.

**Table 5:** BLASTN comparison of the reference sequences of the abundant nucleic acid clusters (>10 and >100 sequences per cluster) from the current study to the total combined mRNA and DNA sequences from the Frias-Lopez et al [9] study.

		Frias-Lopez et al [9] study	
		mRNA homologues (%)	DNA homologues (%)
Current Study	3639 nucleotide clusters (10–99 sequences)	107 (2.9%)	326 (9%)
	85 nucleotide clusters (>100 sequences)	1 (1.2%)	4 (4.7%)

The 3649 clusters have >10 sequences and the 85 clusters are ‘contigs’ of all 609 clusters with >100 sequences (as described in the Materials and Methods).

About 10% of the sequences in the two metagenomes are shared, but the shared proportion of DNA pORFs is higher (15%) for the Frias-Lopez study and considerably lower (3.7%) for the current study. Interestingly, this trend is confirmed by the DNA-mRNA comparisons in which the total proportion of DNA matches is always far lower than the proportion of mRNA (**Table 4**). At the highest level of assembly, comparisons of pORF clusters reveal that 9% and 7.5% of the mRNAs are shared with the relevant metagenome. Smaller proportions of the pORF mRNAs of each study (5.0% and 0.7%) showed similarities to each other suggesting that different subsets of the “potential metatranscriptome” of the two communities are expressed in the two habitats.

Shared sequences are expected to include housekeeping genes, as suggested by the homology between many of the largest identifiable clusters found in both studies **(** **Table 4** and **Table 6**). Similarities among the mostly highly expressed abundant clusters are still very rare (**Table 5**). This could be a result of niche-specific genes (or post-bloom specific genes in this study) and/or the heavy viral load associated with the collapsing algal bloom conditions for the current study. This viral load hypothesis is potentially confirmed by an observed anomaly seen in **Table 4**. The relative percentage of total nucleic acid comparisons is higher than the comparison between nucleic acid clusters (nuc-clusters) for each analysis except when comparing our mRNA nucleotide clusters (mRNA nuc-clusters). The relative percentage increases from 0.53% to 1.45%, and is seen again when comparing against the Frias-Lopez mRNA following removal of the rRNA, whereby the values are 0.43% increasing to 1.2% (**Table 4**). We hypothesise that this anomaly is caused by the majority of the Frias-Lopez mRNA homolog's being singletons in our mRNA data, hence on clustering, their contribution to the percentage calculation is more significant. This highlights the fact that the abundant sequences in our mRNA data are not abundantly expressed in the Frias-Lopez data, which is to be expected if they are viral sequences.

**Table 6:** Top 10 most abundant annotatable transcripts from the Frias-Lopez *et al.* [9].

PFAM				TIGRFAM				COG			
PFAM ID	Annotation	A	B	TIGRfam ID	Annotation	A	B	COG ID	Annotation	A	B
PF00004	ATPase family associated with various cellular activities (AAA)	13	2	TIGR00485	Translation elongation factor Tu	6	2	COG4585	Signal transduction histidine kinase	0	8
PF01370	NAD dependent epimerase/dehydratase family	4	1	TIGR01242	26S proteasome subunit P45 family	6	1	COG5116	26S proteasome regulatory complex component	0	6
PF03143	Elongation factor Tu C-terminal domain	3	1	TIGR02639	ATP-dependent Clp protease ATP-binding subunit ClpA	6	1	COG0050	GTPases - translation elongation factors	6	2
PF00521	DNA gyrase/topoisomerase IV, subunit A	2	1	TIGR00962	ATP synthase F1, alpha subunit	4	1	COG1222	ATP-dependent 26S proteasome regulatory subunit	0	2
PF03144	Elongation factor Tu domain 2	2	1	TIGR01472	GDP-mannose 4,6-dehydratase	3	1	COG0187	Type IIA topoisomerase (DNA gyrase/topo II, topoisomerase IV), B subunit	4	1
PF00216	Bacterial DNA-binding protein	1	1	TIGR01017	Ribosomal protein S4	2	1	COG0568	DNA-directed RNA polymerase, sigma subunit (sigma70/sigma32)	4	1
PF00101	Ribulose bisphosphate carboxylase, small chain	0	1	TIGR02521	Type IV pilus biogenesis/stability protein PilW	1	1	COG1089	GDP-D-mannose dehydratase	3	1
PF00016	Ribulose bisphosphate carboxylase large chain, catalytic domain	0	1	TIGR00038	translation elongation factor P	0	1	COG0188	Type IIA topoisomerase (DNA gyrase/topo II, topoisomerase IV), A subunit	2	1
PF01106	NifU-like domain	0	1	TIGR00050	RNA methyltransferase, TrmH family, group 1	0	1	COG0206	Cell division GTPase	0	1
PF07719	Tetratricopeptide repeat	0	1	TIGR00065	Cell division protein FtsZ	0	1	COG0278	Glutaredoxin-related protein	0	1

(B). If a homologue was identified in the current study (A) that too is included. Numbers in columns A and B refer to the number of sequences which were assigned this particular protein annotation. Only pORF clusters with >10 non-redundant sequences were included in this analysis.

The number of protein sequences that can be annotated through comparison to PFAM, TIGRfam or COG was approximately 2.5% prior to removal of rRNA sequences and 4.3% following removal. This is far lower than the 36.5% from our study which could be annotated (8.5 fold more) (**Tables 2** **and** **Table S1**). When comparing the annotation of the top 10 most abundant pORF clusters (>10 non-redundant sequences) found in the Frias-Lopez studies (**Table 6**) 6 (by PFAM), 7 (by TIGRfam) and 5 (by COG) clusters are found in both studies as abundant clusters (>100 sequences per pORF cluster). For example, the 1^st^ and 2^nd^ most abundant PFAM annotation for the Frias-Lopez study (PF00004) are the 4^th^ and 10^th^ most abundant PFAM annotation for the current study (**Table 3** **&** **6**).

### Summary

The ability to assess natural metatranscriptomes of complex microbial communities under different environmental conditions represents a significant advance in our ability to link community structure with function and DNA genotypes (sequences) with corresponding phenotypes. The approach presented here expands the available methodologies for assaying metatranscriptomes with >99% enrichment from total RNA (by removal of ribosomal RNA) and demonstrates that changes in expression of transcripts can be observed between time points. The outputs of this study include a large number of novel, highly expressed sequence clusters and confirmation that the majority of these clusters are orphaned and therefore further prove the utility of this approach for use in discovering novel genetic capacity [9]. The computational analyses produced in this study also demonstrates the critical importance of access to public portals, namely CAMERA [20] and SEED [17], [18], for the processing of such vast quantities of complex data.

## Supporting Information

Table S1 Comparison of DNA and mRNA from samples collected by Frias-Lopez et al [9].
(0.04 MB RTF)

Table S2 Information about the 85 most abundant nucleotide clusters. Including size, number of sequences in cluster, distribution of abundance of mRNA and DNA sequences within each cluster and the presence or absence of those clusters for which PCR amplification from environmental DNA was performed. T1B1 refers to high CO2 from the mid-bloom; T1B6 refers to present day CO2 from the mid-bloom; T2B1 refers to high CO2 from the post-bloom; T2B6 refers to present day CO2 from the post-bloom.
(0.27 MB RTF)

Table S3 Number of (A) nucleotide sequence and (C) partial ORF sequence homologues found between the eight datasets from the current study. Percentage of (B) nucleotide sequence and (D) partial ORF sequence homologues found between the eight datasets from the current study. T1B1 = Mid-Bloom, High CO2. T1B6 = Mid-Bloom, Present Day. T2B1 = Post-Bloom, High CO2. T2B6 = Post-Bloom, Present Day. pORF percentages are based on total pORFs, denoted d in Table 2.
(0.10 MB RTF)
