# Supplementary material for: Detection of Large Numbers of Novel Sequences in the Metatranscriptomes of Complex Marine Microbial Communities
Source: PLoS One. 2008 Aug 22;3(8):e3042. doi: 10.1371/journal.pone.0003042 (PMC2518522; doi:10.1371/journal.pone.0003042)
Supplement: Table S1 — Comparison of DNA and mRNA from samples collected by Frias-Lopez et al [9]. (0.04 MB RTF) [file pone.0003042.s001.doc]

**Table S1**. Comparison of DNA and mRNA from samples collected by Frias-Lopez et al [9].

|  | DNA | mRNA | All |
| --- | --- | --- | --- |
| Total Size (Mbp) | 45,400,00 | 14,700,000 | 60,100,000 |
| Total No. of reads | 414,323 | 128,234 | 542,557 |
| Average Length (bp) | 110 | 114 | 112 |
| % of rRNA genes ^a^ | 0.59 | 50.8 | 12.5 |
| Absolute number of unique nucleotide clusters ^b^ | 334,940 | 69,948 | 403,665 |
| Total number of reads in top cluster | 14 | 469 | 470 |
| Clustering: 1 sequence | 283401 | **54245** | 336090 |
| 2-9 sequences | 51476 | **14485** | 66267 |
| 10-99 sequences | 63 | **1185** | 1275 |
| 100+ sequences | 0 | **33** | 33 |
| Total pORFs ^c^ | 545,414 | 179,598 | 725,012 |
| Unique pORFs at 95%^d^ | 461,443 | 119,832 | 579,831 |
| Protein clusters ^e^ | 390,599 | 46,703 | 433,892 |
| Protein clusters ^f^ with similarity to: | 24 | 1,786 | 1,826 |
| PFAM ^g^ | 6 | 5 | 13 |
| TIGRfam ^h^ | 7 | 11 | 20 |
| COG ^i^ | 7 | 26 | 36 |
| Number of novel Protein clusters ^j^ | 14 | 1,754 | 1,781 |

^a^Analysis of sequences against the Ribosomal Database Project II (RDP-II) and the European Ribosomal large subunit (LSU)dataset.

^b^ Based on clustering at 95% identity over 80% length of a sequence and over 120 bp.

^c^ Partial Open Reading Frames (pORFs) from six reading frame translation from all reads using translation table 11, starting at the beginning of a read or first ATG after previous stop codon, ending at the end of a read, or at a stop codon and being at least 30 contiguous amino acids.

^d^Total pORF reads clustered at 95% identity of over 80% length of sequences.

^e^ Clusters are identified using the representative sequences of each cluster from the 95% step to cluster at 60% identity of over 80% length of sequences.

^f^ The dominant clusters (≥10 non-redundant sequences) with the exclusion of spurious pORFs.

^g^ Protein families database.

^h^ The Institute for Genomic Research protein database.

^i^ NCBI clusters of orthologous groups database.

^j^ With ≥ 10 non-redundant clustered sequences excluding spurious ORFs.
