# Supplementary material for: Detection of Large Numbers of Novel Sequences in the Metatranscriptomes of Complex Marine Microbial Communities
Source: PLoS One. 2008 Aug 22;3(8):e3042. doi: 10.1371/journal.pone.0003042 (PMC2518522; doi:10.1371/journal.pone.0003042)
Supplement: Table S2 — Information about the 85 most abundant nucleotide clusters. Including size, number of sequences in cluster, distribution of abundance of mRNA and DNA sequences within each cluster and the presence or absence of those clusters for which PCR amplification from environmental DNA was performed. T1B1 refers to high CO2 from the mid-bloom; T1B6 refers to present day CO2 from the mid-bloom; T2B1 refers to high CO2 from the post-bloom; T2B6 refers to present day CO2 from the post-bloom. (0.27 MB RTF) [file pone.0003042.s002.doc]

**Table S2** – Information about the 85 most abundant nucleotide clusters. Including size, number of sequences in cluster, distribution of abundance of mRNA and DNA sequences within each cluster and the presence or absence of those clusters for which PCR amplification from environmental DNA was performed. T1B1 refers to high CO_2_ from the mid-bloom; T1B6 refers to present day CO_2_ from the mid-bloom; T2B1 refers to high CO_2_ from the post-bloom; T2B6 refers to present day CO_2_ from the post-bloom.

| | **ID** | **Number of sequences in cluster** | **Distribution of abundance mRNA sequences** | | | | **Distribution of DNA sequences within clusters** | | | | **Presence/absence of amplified transcripts from environmental DNA** | | | | | --- | --- | --- | --- | --- | --- | --- | --- | --- | --- | --- | --- | --- | --- | | **T1B1** | **T1B6** | **T2B1** | **T2B6** | **T1B1** | **T1B6** | **T2B1** | **T2B6** | **T1B1** | **T1B6** | **T2B1** | **T2B6** | | 1 | 31642 | 2149 | 3054 | 12568 | 13871 | 0 | 0 | 0 | 0 |  |  |  |  | | 2 | 21593 | 1437 | 2136 | 8097 | 9923 | 0 | 0 | 0 | 0 |  |  |  |  | | 3 | 17242 | 1128 | 1687 | 6813 | 7614 | 0 | 0 | 0 | 0 |  |  |  |  | | 4 | 16135 | 7033 | 5478 | 1378 | 2245 | 0 | 0 | 1 | 0 | 1 | 1 | 1 | 1 | | 5 | 14981 | 1033 | 1468 | 5567 | 6912 | 0 | 0 | 0 | 1 |  |  |  |  | | 6 | 14640 | 6446 | 4919 | 1219 | 2056 | 0 | 0 | 0 | 0 | 0 | 0 | 1 | 1 | | 7 | 12597 | 829 | 1238 | 4823 | 5707 | 0 | 0 | 0 | 0 |  |  |  |  | | 8 | 11984 | 698 | 1049 | 4641 | 5595 | 0 | 1 | 0 | 0 |  |  |  |  | | 9 | 10666 | 4597 | 3748 | 881 | 1439 | 1 | 0 | 0 | 0 | 1 | 0 | 1 | 1 | | 10 | 8911 | 564 | 1030 | 3499 | 3818 | 0 | 0 | 0 | 0 |  |  |  |  | | 11 | 8291 | 445 | 636 | 3361 | 3849 | 0 | 0 | 0 | 0 | 0 | 1 | 1 | 1 | | 12 | 8049 | 449 | 557 | 3370 | 3673 | 0 | 0 | 0 | 0 |  |  |  |  | | 13 | 8041 | 531 | 742 | 3070 | 3698 | 0 | 0 | 0 | 0 |  |  |  |  | | 14 | 7286 | 2992 | 2475 | 761 | 1058 | 0 | 0 | 0 | 0 | 1 | 0 | 1 | 1 | | 15 | 6146 | 2740 | 2039 | 519 | 848 | 0 | 0 | 0 | 0 | 0 | 0 | 1 | 1 | | 16 | 5457 | 2471 | 1806 | 447 | 733 | 0 | 0 | 0 | 0 | 1 | 0 | 1 | 1 | | 17 | 5188 | 294 | 353 | 2166 | 2375 | 0 | 0 | 0 | 0 |  |  |  |  | | 18 | 4946 | 255 | 479 | 2000 | 2212 | 0 | 0 | 0 | 0 |  |  |  |  | | 19 | 4542 | 510 | 3855 | 36 | 141 | 0 | 0 | 0 | 0 |  |  |  |  | | 20 | 4458 | 216 | 388 | 1877 | 1977 | 0 | 0 | 0 | 0 |  |  |  |  | | 21 | 4287 | 442 | 3703 | 20 | 122 | 0 | 0 | 0 | 0 |  |  |  |  | | 22 | 4119 | 226 | 377 | 1625 | 1891 | 0 | 0 | 0 | 0 |  |  |  |  | | 23 | 4032 | 231 | 344 | 1621 | 1836 | 0 | 0 | 0 | 0 |  |  |  |  | | 24 | 3245 | 173 | 270 | 1388 | 1413 | 0 | 0 | 1 | 0 |  |  |  |  | | 25 | 2868 | 1247 | 969 | 256 | 396 | 0 | 0 | 0 | 0 |  |  |  |  | | 26 | 2770 | 1105 | 981 | 272 | 412 | 0 | 0 | 0 | 0 |  |  |  |  | | 27 | 2408 | 1084 | 824 | 191 | 309 | 0 | 0 | 0 | 0 | 0 | 0 | 1 | 1 | | 28 | 2267 | 153 | 200 | 805 | 1109 | 0 | 0 | 0 | 0 |  |  |  |  | | 29 | 2039 | 210 | 1556 | 112 | 161 | 0 | 0 | 0 | 0 |  |  |  |  | | 30 | 1723 | 772 | 572 | 146 | 233 | 0 | 0 | 0 | 0 |  |  |  |  | | 31 | 1502 | 164 | 1285 | 13 | 40 | 0 | 0 | 0 | 0 |  |  |  |  | | 32 | 1309 | 16 | 1054 | 58 | 181 | 0 | 0 | 0 | 0 | 1 | 0 | 1 | 1 | | 33 | 1280 | 11 | 1221 | 11 | 37 | 0 | 0 | 0 | 0 |  |  |  |  | | 34 | 1138 | 167 | 129 | 390 | 452 | 0 | 0 | 0 | 0 |  |  |  |  | | 35 | 1102 | 41 | 0 | 505 | 556 | 0 | 0 | 0 | 0 | 0 | 1 | 1 | 1 | | 36 | 1056 | 102 | 63 | 403 | 488 | 0 | 0 | 0 | 0 |  |  |  |  | | 37 | 1047 | 434 | 364 | 111 | 138 | 0 | 0 | 0 | 0 |  |  |  |  | | 38 | 998 | 31 | 1 | 450 | 516 | 0 | 0 | 0 | 0 |  |  |  |  | | 39 | 996 | 69 | 24 | 424 | 479 | 0 | 0 | 0 | 0 |  |  |  |  | | 40 | 980 | 19 | 824 | 35 | 102 | 0 | 0 | 0 | 0 | 0 | 1 | 1 | 1 | | 41 | 978 | 42 | 6 | 428 | 502 | 0 | 0 | 0 | 0 |  |  |  |  | | 42 | 957 | 82 | 847 | 6 | 22 | 0 | 0 | 0 | 0 |  |  |  |  | | 43 | 911 | 80 | 48 | 365 | 418 | 0 | 0 | 0 | 0 | 1 | 0 | 1 | 1 | | 44 | 869 | 32 | 414 | 100 | 323 | 0 | 0 | 0 | 0 | 0 | 1 | 1 | 1 | | 45 | 862 | 8 | 817 | 9 | 28 | 0 | 0 | 0 | 0 | 1 | 0 | 1 | 1 | | 46 | 861 | 171 | 125 | 269 | 296 | 0 | 0 | 0 | 0 |  |  |  |  | | 47 | 857 | 30 | 404 | 102 | 321 | 0 | 0 | 0 | 0 |  |  |  |  | | 48 | 846 | 45 | 69 | 336 | 396 | 0 | 0 | 0 | 0 |  |  |  |  | | 49 | 774 | 25 | 345 | 99 | 305 | 0 | 0 | 0 | 0 | 1 | 1 | 1 | 1 | | 50 | 765 | 107 | 625 | 4 | 29 | 0 | 0 | 0 | 0 |  |  |  |  | | 51 | 722 | 85 | 78 | 265 | 294 | 0 | 0 | 0 | 0 |  |  |  |  | | 52 | 691 | 31 | 639 | 6 | 15 | 0 | 0 | 0 | 0 |  |  |  |  | | 53 | 628 | 29 | 50 | 260 | 289 | 0 | 0 | 0 | 0 |  |  |  |  | | 54 | 626 | 3 | 609 | 6 | 8 | 0 | 0 | 0 | 0 | 1 | 0 | 1 | 1 | | 55 | 562 | 1 | 537 | 5 | 19 | 0 | 0 | 0 | 0 |  |  |  |  | | 56 | 553 | 12 | 315 | 73 | 153 | 0 | 0 | 0 | 0 | 0 | 0 | 1 | 1 | | 57 | 528 | 25 | 498 | 0 | 5 | 0 | 0 | 0 | 0 |  |  |  |  | | 58 | 499 | 5 | 470 | 3 | 21 | 0 | 0 | 0 | 0 | 1 | 0 | 1 | 1 | | 59 | 453 | 14 | 1 | 192 | 246 | 0 | 0 | 0 | 0 | 0 | 1 | 1 | 1 | | 60 | 447 | 8 | 385 | 12 | 42 | 0 | 0 | 0 | 0 |  |  |  |  | | 61 | 441 | 102 | 58 | 136 | 145 | 0 | 0 | 0 | 0 |  |  |  |  | | 62 | 420 | 14 | 0 | 199 | 207 | 0 | 0 | 0 | 0 |  |  |  |  | | 63 | 402 | 1 | 388 | 7 | 6 | 0 | 0 | 0 | 0 | 0 | 1 | 1 | 1 | | 64 | 397 | 5 | 364 | 4 | 24 | 0 | 0 | 0 | 0 | 0 | 0 | 1 | 1 | | 65 | 359 | 2 | 353 | 3 | 1 | 0 | 0 | 0 | 0 |  |  |  |  | | 66 | 356 | 43 | 21 | 143 | 149 | 0 | 0 | 0 | 0 |  |  |  |  | | 67 | 346 | 13 | 1 | 149 | 183 | 0 | 0 | 0 | 0 | 0 | 0 | 1 | 1 | | 68 | 333 | 6 | 169 | 44 | 114 | 0 | 0 | 0 | 0 | 0 | 1 | 1 | 1 | | 69 | 312 | 149 | 92 | 28 | 43 | 0 | 0 | 0 | 0 | 1 | 1 | 1 | 1 | | 70 | 280 | 2 | 265 | 3 | 10 | 0 | 0 | 0 | 0 |  |  |  |  | | 71 | 268 | 0 | 259 | 8 | 1 | 0 | 0 | 0 | 0 |  |  |  |  | | 72 | 261 | 7 | 253 | 1 | 0 | 0 | 0 | 0 | 0 | 1 | 0 | 1 | 1 | | 73 | 230 | 12 | 23 | 105 | 90 | 0 | 0 | 0 | 0 |  |  |  |  | | 74 | 206 | 95 | 67 | 15 | 29 | 0 | 0 | 0 | 0 |  |  |  |  | | 75 | 205 | 78 | 67 | 27 | 33 | 0 | 0 | 0 | 0 |  |  |  |  | | 76 | 201 | 10 | 0 | 96 | 95 | 0 | 0 | 0 | 0 | 0 | 0 | 1 | 1 | | 77 | 132 | 53 | 47 | 10 | 22 | 0 | 0 | 0 | 0 |  |  |  |  | | 78 | 125 | 31 | 22 | 38 | 34 | 0 | 0 | 0 | 0 |  |  |  |  | | 79 | 117 | 9 | 104 | 2 | 2 | 0 | 0 | 0 | 0 |  |  |  |  | | 80 | 115 | 46 | 66 | 2 | 1 | 0 | 0 | 0 | 0 |  |  |  |  | | 81 | 112 | 43 | 62 | 2 | 5 | 0 | 0 | 0 | 0 |  |  |  |  | | 82 | 112 | 40 | 66 | 1 | 5 | 0 | 0 | 0 | 0 | 0 | 1 | 1 | 1 | | 83 | 105 | 19 | 83 | 3 | 0 | 0 | 0 | 0 | 0 |  |  |  |  | | 84 | 105 | 37 | 41 | 12 | 15 | 0 | 0 | 0 | 0 |  |  |  |  | | 85 | 105 | 61 | 25 | 9 | 10 | 0 | 0 | 0 | 0 |  |  |  |  | |
| --- | --- | --- | --- | --- | --- | --- | --- | --- | --- | --- | --- | --- | --- | --- | --- | --- | --- | --- | --- | --- | --- | --- | --- | --- | --- | --- | --- | --- | --- | --- | --- | --- | --- | --- | --- | --- | --- | --- | --- | --- | --- | --- | --- | --- | --- | --- | --- | --- | --- | --- | --- | --- | --- | --- | --- | --- | --- | --- | --- | --- | --- | --- | --- | --- | --- | --- | --- | --- | --- | --- | --- | --- | --- | --- | --- | --- | --- | --- | --- | --- | --- | --- | --- | --- | --- | --- | --- | --- | --- | --- | --- | --- | --- | --- | --- | --- | --- | --- | --- | --- | --- | --- | --- | --- | --- | --- | --- | --- | --- | --- | --- | --- | --- | --- | --- | --- | --- | --- | --- | --- | --- | --- | --- | --- | --- | --- | --- | --- | --- | --- | --- | --- | --- | --- | --- | --- | --- | --- | --- | --- | --- | --- | --- | --- | --- | --- | --- | --- | --- | --- | --- | --- | --- | --- | --- | --- | --- | --- | --- | --- | --- | --- | --- | --- | --- | --- | --- | --- | --- | --- | --- | --- | --- | --- | --- | --- | --- | --- | --- | --- | --- | --- | --- | --- | --- | --- | --- | --- | --- | --- | --- | --- | --- | --- | --- | --- | --- | --- | --- | --- | --- | --- | --- | --- | --- | --- | --- | --- | --- | --- | --- | --- | --- | --- | --- | --- | --- | --- | --- | --- | --- | --- | --- | --- | --- | --- | --- | --- | --- | --- | --- | --- | --- | --- | --- | --- | --- | --- | --- | --- | --- | --- | --- | --- | --- | --- | --- | --- | --- | --- | --- | --- | --- | --- | --- | --- | --- | --- | --- | --- | --- | --- | --- | --- | --- | --- | --- | --- | --- | --- | --- | --- | --- | --- | --- | --- | --- | --- | --- | --- | --- | --- | --- | --- | --- | --- | --- | --- | --- | --- | --- | --- | --- | --- | --- | --- | --- | --- | --- | --- | --- | --- | --- | --- | --- | --- | --- | --- | --- | --- | --- | --- | --- | --- | --- | --- | --- | --- | --- | --- | --- | --- | --- | --- | --- | --- | --- | --- | --- | --- | --- | --- | --- | --- | --- | --- | --- | --- | --- | --- | --- | --- | --- | --- | --- | --- | --- | --- | --- | --- | --- | --- | --- | --- | --- | --- | --- | --- | --- | --- | --- | --- | --- | --- | --- | --- | --- | --- | --- | --- | --- | --- | --- | --- | --- | --- | --- | --- | --- | --- | --- | --- | --- | --- | --- | --- | --- | --- | --- | --- | --- | --- | --- | --- | --- | --- | --- | --- | --- | --- | --- | --- | --- | --- | --- | --- | --- | --- | --- | --- | --- | --- | --- | --- | --- | --- | --- | --- | --- | --- | --- | --- | --- | --- | --- | --- | --- | --- | --- | --- | --- | --- | --- | --- | --- | --- | --- | --- | --- | --- | --- | --- | --- | --- | --- | --- | --- | --- | --- | --- | --- | --- | --- | --- | --- | --- | --- | --- | --- | --- | --- | --- | --- | --- | --- | --- | --- | --- | --- | --- | --- | --- | --- | --- | --- | --- | --- | --- | --- | --- | --- | --- | --- | --- | --- | --- | --- | --- | --- | --- | --- | --- | --- | --- | --- | --- | --- | --- | --- | --- | --- | --- | --- | --- | --- | --- | --- | --- | --- | --- | --- | --- | --- | --- | --- | --- | --- | --- | --- | --- | --- | --- | --- | --- | --- | --- | --- | --- | --- | --- | --- | --- | --- | --- | --- | --- | --- | --- | --- | --- | --- | --- | --- | --- | --- | --- | --- | --- | --- | --- | --- | --- | --- | --- | --- | --- | --- | --- | --- | --- | --- | --- | --- | --- | --- | --- | --- | --- | --- | --- | --- | --- | --- | --- | --- | --- | --- | --- | --- | --- | --- | --- | --- | --- | --- | --- | --- | --- | --- | --- | --- | --- | --- | --- | --- | --- | --- | --- | --- | --- | --- | --- | --- | --- | --- | --- | --- | --- | --- | --- | --- | --- | --- | --- | --- | --- | --- | --- | --- | --- | --- | --- | --- | --- | --- | --- | --- | --- | --- | --- | --- | --- | --- | --- | --- | --- | --- | --- | --- | --- | --- | --- | --- | --- | --- | --- | --- | --- | --- | --- | --- | --- | --- | --- | --- | --- | --- | --- | --- | --- | --- | --- | --- | --- | --- | --- | --- | --- | --- | --- | --- | --- | --- | --- | --- | --- | --- | --- | --- | --- | --- | --- | --- | --- | --- | --- | --- | --- | --- | --- | --- | --- | --- | --- | --- | --- | --- | --- | --- | --- | --- | --- | --- | --- | --- | --- | --- | --- | --- | --- | --- | --- | --- | --- | --- | --- | --- | --- | --- | --- | --- | --- | --- | --- | --- | --- | --- | --- | --- | --- | --- | --- | --- | --- | --- | --- | --- | --- | --- | --- | --- | --- | --- | --- | --- | --- | --- | --- | --- | --- | --- | --- | --- | --- | --- | --- | --- | --- | --- | --- | --- | --- | --- | --- | --- | --- | --- | --- | --- | --- | --- | --- | --- | --- | --- | --- | --- | --- | --- | --- | --- | --- | --- | --- | --- | --- | --- | --- | --- | --- | --- | --- | --- | --- | --- | --- | --- | --- | --- | --- | --- | --- | --- | --- | --- | --- | --- | --- | --- | --- | --- | --- | --- | --- | --- | --- | --- | --- | --- | --- | --- | --- | --- | --- | --- | --- | --- | --- | --- | --- | --- | --- | --- | --- | --- | --- | --- | --- | --- | --- | --- | --- | --- | --- | --- | --- | --- | --- | --- | --- | --- | --- | --- | --- | --- | --- | --- | --- | --- | --- | --- | --- | --- | --- | --- | --- | --- | --- | --- | --- | --- | --- | --- | --- | --- | --- | --- | --- | --- | --- | --- | --- | --- | --- | --- | --- | --- | --- | --- | --- | --- | --- | --- | --- | --- | --- | --- | --- | --- | --- | --- | --- | --- | --- | --- | --- | --- | --- | --- | --- | --- | --- | --- | --- | --- | --- | --- | --- | --- | --- | --- | --- | --- | --- | --- | --- | --- | --- | --- | --- | --- | --- | --- | --- | --- | --- | --- | --- | --- | --- | --- | --- | --- | --- | --- | --- | --- | --- | --- | --- | --- | --- | --- | --- | --- | --- | --- | --- | --- | --- | --- | --- | --- | --- | --- | --- | --- | --- | --- | --- | --- | --- | --- | --- | --- | --- | --- | --- | --- | --- | --- | --- | --- | --- | --- | --- | --- | --- | --- | --- | --- | --- | --- | --- | --- | --- | --- | --- | --- | --- | --- | --- | --- | --- | --- | --- | --- | --- | --- | --- | --- | --- | --- | --- | --- | --- | --- | --- | --- | --- | --- | --- | --- | --- | --- | --- | --- | --- | --- | --- | --- | --- | --- | --- | --- | --- | --- | --- | --- | --- | --- | --- | --- | --- | --- | --- | --- | --- | --- | --- | --- | --- | --- | --- | --- | --- | --- | --- | --- | --- | --- | --- | --- | --- | --- | --- | --- | --- | --- | --- | --- | --- | --- | --- | --- | --- | --- | --- | --- | --- | --- | --- | --- | --- | --- | --- | --- | --- | --- | --- | --- | --- | --- | --- | --- | --- | --- | --- | --- | --- | --- | --- | --- | --- | --- | --- | --- | --- | --- | --- | --- | --- | --- | --- | --- | --- | --- | --- | --- | --- | --- | --- | --- | --- | --- | --- | --- | --- | --- | --- | --- | --- | --- | --- | --- | --- | --- | --- | --- | --- | --- | --- | --- | --- | --- | --- | --- | --- | --- | --- | --- | --- | --- | --- | --- | --- | --- | --- | --- | --- | --- | --- | --- | --- | --- | --- | --- | --- | --- | --- | --- | --- | --- | --- | --- | --- | --- | --- | --- | --- | --- | --- | --- | --- | --- | --- | --- | --- | --- | --- | --- | --- | --- | --- | --- | --- | --- | --- | --- | --- | --- | --- | --- | --- | --- | --- | --- | --- | --- | --- | --- | --- | --- | --- | --- | --- |
