# Supplementary material for: Detection of Large Numbers of Novel Sequences in the Metatranscriptomes of Complex Marine Microbial Communities
Source: PLoS One. 2008 Aug 22;3(8):e3042. doi: 10.1371/journal.pone.0003042 (PMC2518522; doi:10.1371/journal.pone.0003042)
Supplement: Table S3 — Number of (A) nucleotide sequence and (C) partial ORF sequence homologues found between the eight datasets from the current study. Percentage of (B) nucleotide sequence and (D) partial ORF sequence homologues found between the eight datasets from the current study. T1B1 = Mid-Bloom, High CO2. T1B6 = Mid-Bloom, Present Day. T2B1 = Post-Bloom, High CO2. T2B6 = Post-Bloom, Present Day. pORF percentages are based on total pORFs, denoted d in Table 2. (0.10 MB RTF) [file pone.0003042.s003.doc]

**Table S3.** Number of (A) nucleotide sequence and (C) partial ORF sequence homologues found between the eight datasets from the current study. Percentage of (B) nucleotide sequence and (D) partial ORF sequence homologues found between the eight datasets from the current study. T1B1 = Mid-Bloom, High CO_2_. T1B6 = Mid-Bloom, Present Day. T2B1 = Post-Bloom, High CO_2_. T2B6 = Post-Bloom, Present Day. pORF percentages are based on total pORFs, denoted ^d^ in Table 2.

**A**

|  | DNA_T1B1 | DNA_T1B6 | DNA_T2B1 | DNA_T2B6 | mRNA_T1B1 | mRNA_T1B6 | mRNA_T2B1 | mRNA_T2B6 |
| --- | --- | --- | --- | --- | --- | --- | --- | --- |
| DNA_T1B1 | 209073:209073 | 26442:24107 | 44840:51724 | 42268:45797 | 15393:13117 | 13063:11407 | 1168:855 | 1135:870 |
| DNA_T1B6 | 24107:26442 | 134915:134915 | 28405:35795 | 28304:33780 | 8919:8318 | 7924:7688 | 739:680 | 649:589 |
| DNA_T2B1 | 51724:44840 | 35795:28405 | 344216:344216 | 76566:72201 | 18202:14055 | 17063:13437 | 2406:1569 | 2278:1574 |
| DNA_T2B6 | 45797:42268 | 33780:28304 | 72201:76566 | 304020:304020 | 16270:13088 | 14798:12191 | 1756:1338 | 1552:1295 |
| mRNA_T1B1 | 13117:15393 | 8318:8919 | 14055:18202 | 13088:16270 | 131089:131089 | 70330:93288 | 54449:87161 | 56433:105215 |
| mRNA_T1B6 | 11407:13063 | 7688:7924 | 13437:17063 | 12191:14798 | 93288:70330 | 162871:162871 | 75012:85859 | 78874:104649 |
| mRNA_T2B1 | 855:1168 | 680:739 | 1569:2406 | 1338:1756 | 87161:54449 | 85859:75012 | 96201:96201 | 88613:106379 |
| mRNA_T2B6 | 870:1135 | 589:649 | 1574:2278 | 1295:1552 | 105215:56433 | 104649:78874 | 106379:88613 | 116192:116192 |

B

|  | DNA_T1B1 | DNA_T1B6 | DNA_T2B1 | DNA_T2B6 | mRNA_T1B1 | mRNA_T1B6 | mRNA_T2B1 | mRNA_T2B6 |
| --- | --- | --- | --- | --- | --- | --- | --- | --- |
| DNA_T1B1 | 100:100 | 12.6:17.9 | 21.4:15.0 | 20.2:15.0 | 7.4:10.0 | 6.2:7.0 | 0.6:0.9 | 0.5:0.7 |
| DNA_T1B6 | 17.9:12.6 | 100:100 | 21.0:10.4 | 21.0:11.1 | 6.6:6.3 | 5.9:4.7 | 0.5:0.7 | 0.5:0.5 |
| DNA_T2B1 | 15.0:21.4 | 10.4:21.0 | 100:100 | 22.2:23.7 | 5.3:10.7 | 5.0:8.3 | 0.7:1.6 | 0.4:1.4 |
| DNA_T2B6 | 15.0:20.2 | 11.1:21.0 | 23.7:22.2 | 100:100 | 5.3:10.0 | 4.9:7.5 | 0.6:1.4 | 0.5:1.1 |
| mRNA_T1B1 | 10.0:7.4 | 6.3:6.6 | 10.7:5.3 | 10.0:5.3 | 100:100 | 53.7:57.2 | 41.9:90.0 | 43.0:90.5 |
| mRNA_T1B6 | 7.0:6.2 | 4.7:5.9 | 8.3:5.0 | 7.5:4.9 | 57.2:53.7 | 100:100 | 46.0:89.2 | 48.4:90.0 |
| mRNA_T2B1 | 0.9:0.6 | 0.7:0.5 | 1.6:0.7 | 1.4:0.6 | 90.0:41.9 | 89.2:46.0 | 100:100 | 92.1:91.4 |
| mRNA_T2B6 | 0.7:0.5 | 0.5:0.5 | 1.4:0.4 | 1.1:0.5 | 90.5:43.0 | 90.0:48.4 | 91.4:92.1 | 100:100 |

C

|  | DNA_T1B1 | DNA_T1B6 | DNA_T2B1 | DNA_T2B6 | mRNA_T1B1 | mRNA_T1B6 | mRNA_T2B1 | mRNA_T2B6 |
| --- | --- | --- | --- | --- | --- | --- | --- | --- |
| DNA_T1B1 | 419565:419565 | 91782:77620 | 116368:133240 | 132595:156454 | 51845:41475 | 45127:36828 | 5368:3420 | 5099:3938 |
| DNA_T1B6 | 77620:91782 | 279061:279061 | 76870:104349 | 91062:127288 | 31659:27618 | 28700:25539 | 3485:2141 | 3398:2176 |
| DNA_T2B1 | 133240:116368 | 104349:76870 | 532373:532373 | 179628:187554 | 53823:36560 | 50783:35761 | 7986:4879 | 7717:5140 |
| DNA_T2B6 | 156454:132595 | 127288:91062 | 187554:179628 | 637896:637896 | 61194:39931 | 56436:38002 | 8009:5549 | 7258:5682 |
| mRNA_T1B1 | 41475:51845 | 27618:31659 | 36560:53823 | 39931:61194 | 284665:284665 | 177321:225190 | 139228:222617 | 143820:271791 |
| mRNA_T1B6 | 36828:45127 | 25539:28700 | 35761:50783 | 38002:56436 | 225190:177321 | 345502:345502 | 182837:221279 | 192412:271659 |
| mRNA_T2B1 | 3420:5368 | 2141:3485 | 4879:7986 | 5549:8009 | 222617:139228 | 221279:182837 | 237187:237187 | 224376:272936 |
| mRNA_T2B6 | 3938:5099 | 2176:3398 | 5140:7717 | 5682:7258 | 271791:143820 | 271659:192412 | 272936:224376 | 289951:289951 |

**D**

|  | DNA_T1B1 | DNA_T1B6 | DNA_T2B1 | DNA_T2B6 | mRNA_T1B1 | mRNA_T1B6 | mRNA_T2B1 | mRNA_T2B6 |
| --- | --- | --- | --- | --- | --- | --- | --- | --- |
| DNA_T1B1 | 100:100 | 21.9:27.80 | 27.7:25.0 | 31.6:24.5 | 12.3:14.5 | 10.8:10.7 | 1.3:1.4 | 1.2:1.4 |
| DNA_T1B6 | 27.8:21.9 | 100:100 | 27.5:19.6 | 32.6:20.0 | 11.3:9.7 | 10.3:7.4 | 1.2:0.9 | 1.2:0.8 |
| DNA_T2B1 | 25.0:27.7 | 19.6:27.5 | 100:100 | 33.7:29.4 | 10.1:12.8 | 9.5:10.4 | 1.5:2.1 | 1.4:1.8 |
| DNA_T2B6 | 24.5:31.6 | 20.0:32.6 | 29.4:33.7 | 100:100 | 9.6:14.0 | 8.8:11.0 | 1.3:2.3 | 1.1:2.0 |
| mRNA_T1B1 | 14.5:12.3 | 9.7:11.3 | 12.8:10.1 | 14.0:9.6 | 100:100 | 62.3:65.2 | 49.0:93.9 | 50.5:93.7 |
| mRNA_T1B6 | 10.7:10.8 | 7.4:10.3 | 10.4:9.5 | 11.0:8.8 | 65.2:62.3 | 100:100 | 53.0:93.3 | 55.7:93.7 |
| mRNA_T2B1 | 1.4:1.3 | 0.9:1.2 | 2.1:1.5 | 2.3:1.3 | 93.9:49.0 | 93.3:53.0 | 100:100 | 94.6:94.1 |
| mRNA_T2B6 | 1.4:1.2 | 0.8:1.2 | 1.8:1.4 | 2.0:1.0 | 93.7:50.5 | 93.7:55.7 | 94.1:94.6 | 100:100 |
